# Frailty and nutritional assessments for predicting postoperative complications in older adults undergoing non-cardiac surgery

**DOI:** 10.3389/fmed.2025.1636091

**Published:** 2025-08-06

**Authors:** Sunisa Chatmongkolchart, Mantana Saetang, Panalee Kittisopaporn, Thitikan Kunapaisal, Dararat Yongsata, Khwanrut Sukitpaneenit

**Affiliations:** Department of Anesthesiology, Faculty of Medicine, Prince of Songkla University, Hat-Yai, Thailand

**Keywords:** Clinical Frailty Scale, malnutrition, older adults, postoperative complications, Prognostic Nutritional Index

## Abstract

**Introduction:**

Frailty and malnutrition are closely linked to adverse surgical outcomes. However, their combined influence on post-non-cardiac surgery complications in older patients remains unclear. We aimed to evaluate the predictive value of frailty, malnutrition, and their combined effect on postoperative complications in older patients undergoing intermediate- to high-risk non-cardiac surgery.

**Methods:**

This study was a retrospective analysis of data from a prospectively collected cohort that included 637 patients aged ≥60 years. We assessed frailty using the Clinical Frailty Scale (CFS) and nutritional status using the Prognostic Nutritional Index (PNI). The primary outcome was the occurrence of postoperative complications (Clavien-Dindo classification ≥2). We used logistic regression and receiver-operating characteristic (ROC) analyses to determine the predictive accuracy of CFS, PNI, and CFS + PNI.

**Results:**

We identified frailty (CFS ≥ 5) in 38.5% of patients, with 29.8% being malnourished (PNI < 45). Multivariate analysis revealed that frailty independently predicted postoperative complications (odds ratio [OR]: 2.09, 95% confidence interval [CI]: 1.09–4.00; *p* = 0.026). Severe malnutrition (PNI < 40) exhibited a strong association with complications in univariate analysis (OR: 5.88, 95%CI: 3.13–11.08; *p* < 0.001) but not in multivariate analysis. The combined CFS + PNI model showed enhanced discriminative ability (area under the curve [AUC]: 0.694, 95%CI: 0.647–0.740) compared with CFS (AUC: 0.619, 95%CI: 0.575–0.662) or PNI (AUC: 0.665, 95%CI: 0.618–0.712) alone.

**Conclusion:**

Frailty is a strong independent predictor of postoperative complications in older patients undergoing non-cardiac surgery. Although severe malnutrition correlates with increased risk, its effect may be partially mediated by frailty. The integration of frailty and nutritional assessments enhances postoperative complication prediction, underscoring comprehensive geriatric evaluation value in preoperative risk stratification.

## 1 Introduction

Age is a primary risk factor for the development of chronic disease; therefore, older individuals are particularly susceptible to disease-related weight loss, loss of muscle mass and strength (i.e., sarcopenia), and, ultimately, frailty syndrome, all of which can profoundly affect recovery from diseases and clinical outcomes (1–3). Consequently, malnutrition and frailty are recognized as two prevalent geriatric syndromes that significantly contribute to adverse outcomes, including disability, dysfunction, falls, and perioperative complications. These conditions can prolong the length of hospital stay (LOS) and increase hospitalization costs, often resulting in the need for long-term care or even mortality (4–6).

Frailty is a clinical syndrome characterized by reduced physiological reserves and heightened vulnerability to stressors. Its prevalence is estimated to range from 18.8 to 41.9% in geriatric surgical patients and from 10.4 to 37.0% in general surgical patients (7, 8). Frailty is associated with decreased functional capacity, an elevated risk of adverse health outcomes, and a higher likelihood of postoperative complications (8).

Additionally, older adults are particularly vulnerable to nutritional deficiencies, which can lead to malnutrition through various mechanisms (9, 10). The reported prevalence of malnutrition is typically below 10% among independently living older adults but increases to nearly two-thirds in hospitalized older patients (11, 12). Malnutrition is linked to adverse outcomes such as higher infection rates, prolonged LOS, delayed recovery after acute illnesses, and increased mortality risk (13, 14). It is also considered an important factor contributing to the multifaceted etiology of frailty (15).

Although frailty and malnutrition have distinct diagnostic criteria, they share common features—such as weight loss and muscle weakness—and often coexist in hospitalized older patients (16). The coexistence of frailty and malnutrition in older surgical patients intensifies their vulnerability to postoperative complications and mortality. However, the interaction between these two factors and their combined influence on surgical outcomes remain inadequately understood. Addressing this gap in knowledge is critical to improving preoperative assessments, optimizing patient care, and enhancing surgical outcomes. In this study, we aimed to evaluate the predictive value of frailty and malnutrition as well as their combined effects on postoperative complications in older patients undergoing surgery.

## 2 Materials and methods

### 2.1 Study design and setting

This study was a retrospective analysis of data derived from a previously approved prospective observational cohort (REC.64-396-8-1) conducted at Songklanagarind Hospital, a tertiary care academic center in Southern Thailand known for its specialized geriatric and surgical services. The hospital operates under Thailand’s Universal Health Coverage system. Most patients were insured through either the Universal Coverage Scheme or the Civil Servant Medical Benefit Scheme, both of which provide equitable access to essential healthcare services regardless of income. All participants received care at this public institution; no patient was treated in private healthcare facilities.

The original cohort was designed to compare the predictive value of three brief frailty assessment tools for postoperative complications in older patients undergoing intermediate- to high-risk non-cardiac surgery. Frailty and nutritional assessments were performed prospectively by trained clinicians as part of routine preoperative evaluations.

In the present analysis, we retrospectively extracted and analyzed this dataset to evaluate the predictive value of frailty (via the Clinical Frailty Scale [CFS]), malnutrition (via the Prognostic Nutritional Index [PNI]), and their combined effect on postoperative complications. The study protocol for this secondary analysis was approved by the Institutional Ethics Committee of the Faculty of Medicine, Prince of Songkla University (Approval Reference: REC.67-484-8-1) on November 11, 2024. The requirement for informed consent was waived owing to the retrospective nature of the analysis. All procedures were conducted in accordance with the Declaration of Helsinki. Consecutive eligible patients were included, and standardized data collection procedures were used to minimize the risk of selection and information bias.

### 2.2 Study participants

The study included patients aged ≥60 years, who were scheduled for elective non-cardiac surgery. Patients undergoing surgeries categorized as intermediate- to high-risk based on the cardiac risk stratification for non-cardiac procedures were eligible for inclusion. Only the first surgery during hospitalization was considered in each patient. Patients deemed too unwell for preoperative assessment and those receiving palliative care were excluded. This study was a secondary analysis of a prospective observational cohort (REC.64–396–8-1) in which both CFS and PNI data were prospectively and systematically collected. Consequently, no patients were excluded owing to missing CFS or PNI data, and no imputation was required.

### 2.3 Outcomes

The study aimed to compare sensitivity, specificity, receiver operating characteristic (ROC) curves, and area under the curve (AUC) to assess predictive accuracy by using frailty tools, nutritional assessment, and a combination of both tools in predicting postoperative complications (Clavien-Dindo classification ≥2) among older patients with intermediate- or high-risk non-cardiac surgery.

We assessed frailty using the CFS (17), which provides a clinical approach for identifying frailty. It consists of nine clinical stages, for which scores of 1–2, 3–4, and ≥5 indicate robust, pre-frail, and frail patients, respectively. All CFS assessments were performed prospectively by two nurse anesthetists who had received standardized training in the use of the CFS before data collection. To minimize inter-rater variability, the training involved structured case-based discussions and calibration sessions using clinical vignettes.

Nutritional status was assessed by calculating the PNI, which is computed as $\;10\times \text{albumin}\;(g/\mathrm{dL})+0.005\times \text{total lymphocyte count}\;(\mathrm{per}\;mm^{3}).$ Nutritional status was divided into four grades based on PNI scores: normal (PNI ≥ 50), mild malnutrition (PNI 45–50), moderate to severe malnutrition (PNI 40–45), and serious malnutrition (PNI < 40) (18, 19). The cutoff value of PNI < 45 was selected to define clinically significant malnutrition, based on previous studies (20, 21) demonstrating its association with adverse nutritional status in surgical populations.

### 2.4 Statistical analysis

We expressed categorical variables as numbers and percentages and compared differences between groups using the Chi-Square or Fisher’s exact test, as appropriate. We expressed continuous variables as mean and standard deviation or median and interquartile range (IQR) and determined differences between groups using the *t* test or rank sum test, as appropriate. We used the Wilcoxon rank-sum test to compare two independent samples. Logistic regression analysis was performed to evaluate the associations of CFS, PNI, and CFS + PNI with postoperative complications.

We constructed a ROC curve to evaluate the discriminatory powers of CFS, PNI, and the combination of both tools for predicting postoperative complications. We calculated the discriminative accuracy using C-statistics from logistic regression, and reported the value as the AUC with 95% confidence intervals (CIs). To compare the performance of the two models, we conducted a pairwise comparison of the ROC curves using the bootstrap test for correlated ROC curves, as implemented in the “pROC” package in R. A total of 2,000 bootstrap replicates were used with stratified resampling. Diagnostic accuracy was defined as unsatisfactory, satisfactory, good, or very good if the AUC was <0.6, 0.6–0.69, 0.7–0.79, or ≥0.8, respectively (22). We used R software (version 4.3.1; R Foundation for Statistical Computing, Vienna, Austria) for statistical analysis, and *p*-values of <0.05 indicated statistical significance.

## 3 Results

### 3.1 Patient characteristics

We included 637 older patients who underwent intermediate- to high-risk non-cardiac surgery in this study. Of these, we classified 38.5% (245 patients) as frail according to the CFS, whereas we identified 29.8% (190 patients) as malnourished based on the PNI. The median PNI was decreased significantly with increasing frailty, from 51.5 in robust individuals to 46 in patients with frailty (*p* < 0.001). Overlap between frailty and malnutrition was observed in 106 patients (16.6%) (Table 1). The median [IQR] age of the patients was the highest in the frail group (73 [67–79] years) compared to the robust (67 [62.2–70.8] years) and pre-frail (68 [64–73] years) groups (*p* < 0.001). Similarly, patients with lower PNI tended to be older (*p* < 0.001). The proportion of women was higher among patients with frailty (61.2%) compared to robust (55.3%) and pre-frail patients (44.9%) (*p* < 0.001). In contrast, men were more likely to be malnourished (53.7%) based on the PNI criteria (*p* = 0.007). Patients with frailty were more likely to be widowed, have lower educational attainment, and require assistance with living compared to robust patients. Similarly, patients with poorer nutritional status (lower PNI scores) tended to have lower education levels and were more likely to live with assistance, although marital status did not significantly differ across nutritional risk groups. Patients with frailty also exhibited a higher prevalence of comorbidities (*p* = 0.048). The prevalence of preoperative anemia increased with both frailty and poor nutritional status (Supplementary Table S1).

**Table 1:** Factors associated with postoperative complications in older patients based on the Clavien-Dindo classification.

Factor-associated postoperative complications	CDC 0–1 (*n* = 471)	CDC ≥2 (*n* = 166)	*p* value
Age (years), median (IQR)	69 (65, 74)	71.5 (65, 78)	0.002
Sex, *n* (%)			<0.001
Male	207 (43.9)	100 (60.2)	
Female	264 (56.1)	66 (39.8)	
Body weight (kg), median (IQR)	61 (53, 69)	59.2 (51, 66)	0.019
Body height (cm), median (IQR)	158 (151, 164)	160 (154, 165)	0.01
BMI (kg/m^2^), median (IQR)	24.3 (21.6, 27.6)	22.7 (20.2, 25.3)	<0.001
PNI, median (IQR)	50 (46, 53)	46 (42, 50)	<0.001
PNI scores			<0.001
<40	32 (51.6)	30 (48.4)	
40–45	81 (63.3)	47 (36.7)	
45–50	142 (72.4)	54 (27.6)	
>50	216 (86.1)	35 (13.9)	
Frail by CFS	152 (32.3)	93 (56)	<0.001
Mid-arm circumference (cm), median (IQR)	27 (24.5, 29.5)	26 (23.5, 28)	<0.001
Albumin level, median (IQR)	4 (3.7, 4.3)	3.8 (3.3, 4)	<0.001
Hb, median (IQR)	12.3 (11.1, 13.2)	11.3 (10.3, 12.6)	<0.001
CrCl, median (IQR)	64 (50, 79.2)	55.7 (43, 71.8)	<0.001
Preoperative anemia, *n* (%)	260 (55.2)	125 (75.3)	<0.001

BMI, body mass index; CDC, Clavien-Dindo classification; CFS, Clinical Frailty Scale; CrCl, creatinine clearance; Hb, hemoglobin; IQR, interquartile range; PNI, Prognostic Nutritional Index.

### 3.2 Postoperative complications

Postoperative complications, according to Clavien-Dindo classification ≥2, were more common in patients with frailty (38%) than in non-frail patients (18.6%) (*p* < 0.001) and in those with PNI scores <45 (40.5%) than in those with PNI scores >45 (19.9%) (*p* < 0.001) (Table 2). Patients with frailty experienced significantly longer LOS than non-frail patients (*p* < 0.001). Similarly, patients with PNI scores <45 had a longer LOS than those with PNI scores >45 (*p* < 0.001). We observed no significant differences in the 30-day mortality between patients with and without frailty (*p* = 0.114). Similarly, no significant differences in mortality were observed between PNI categories (*p* = 0.106) (Supplementary Table S2).

**Table 2:** Univariate and multivariate analyses of factors associated with postoperative complications based on CDC ≥ 2.

	Univariate analysis		Multivariate analysis	
	OR (95%CI)	*p* value	OR (95%CI)	*p* value
Age	1.05 (1.02–1.07)	<0.001	0.98 (0.95–1.02)	0.38
Sex: female vs. male	0.54 (0.37–0.79)	0.001	0.52 (0.27–1)	0.051
BMI	0.92 (0.88–0.96)	<0.001	1.05 (0.96–1.16)	0.279
PNI score: Reference: ≥50				
<40	5.88 (3.13–11.08)	<0.001	1.81 (0.37–8.85)	0.462
40–45	3.93 (2.31–6.69)	<0.001	2.18 (0.74–6.44)	0.157
45–50	2.48 (1.51–4.07)	<0.001	1.85 (0.84–4.06)	0.128
Frail by CFS	2.81 (1.93–4.09)	<0.001	2.09 (1.09–4)	0.026
Mid-arm Circumference	0.9 (0.86–0.95)	<0.001	0.93 (0.84–1.03)	0.158
Albumin level	0.32 (0.22–0.46)	<0.001	0.66 (0.28–1.58)	0.356
Hb	0.81 (0.73–0.9)	<0.001	0.97 (0.82–1.14)	0.688
CrCl	0.99 (0.98–1)	0.003	0.9948 (0.9856–1.004)	0.268
Preoperative Anemia	2.35 (1.56–3.54)	<0.001	1.31 (0.68–2.55)	0.418

BMI, body mass index; CDC, Clavien-Dindo classification; CFS, Clinical Frailty Scale; CI, confidence interval; CrCl, creatinine clearance; Hb, hemoglobin; IQR, interquartile range; OR, odds ratio; PNI, Prognostic Nutritional Index.

Several patient characteristics were found to be significantly associated with increased postoperative complications, including frailty and PNI scores <46 (Table 1).

In multivariate analysis, frailty persisted as an independent predictor of postoperative complications (odds ratio [OR]: 2.09, 95%CI: 1.09–4.00, *p* = 0.026), even after adjusting for other covariates. For malnutrition, a PNI score of 45–50 was associated with a higher risk of complications (OR: 1.85, 95%CI: 0.84–4.06, *p* = 0.128), although this association was not significant. Additionally, a PNI score <40 showed a strong univariate association with complications (OR: 5.88, 95%CI: 3.13–11.08, *p* < 0.001), but this association was not significant in multivariate analysis (OR: 1.81, 95%CI: 0.37–8.85, *p* = 0.462) (Table 2).

### 3.3 ROC curve and predictive accuracy

**Figure 1:**
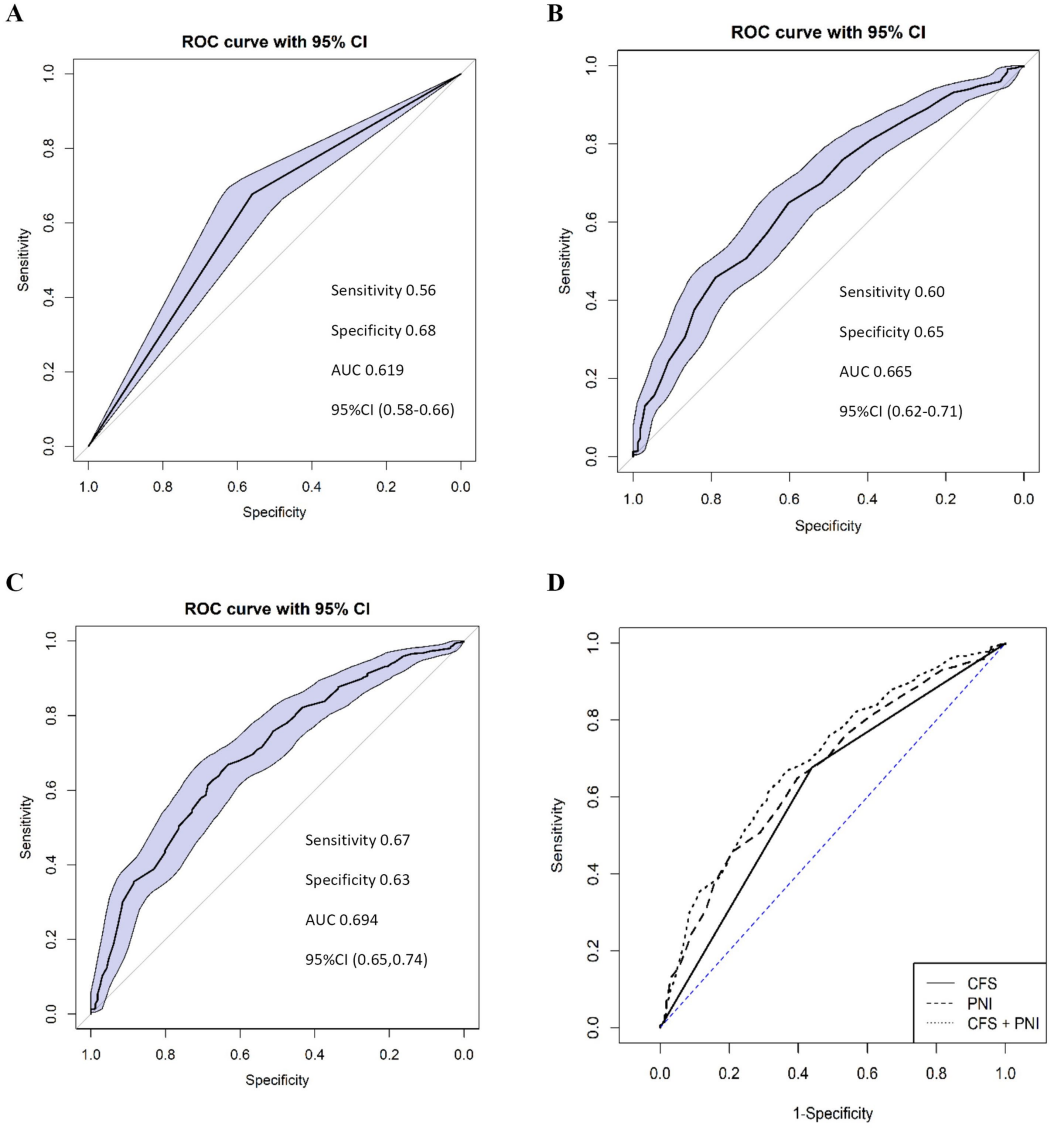
Receiver-operating characteristic (ROC) curve analysis of the CFS **(A)**, PNI **(B)**, and their combination **(C)** to predict postoperative complications (Clavien-Dindo classification ≥2). The combined model **(D)** demonstrates improved predictive performance compared to CFS or PNI alone. CFS **(A)**: AUC = 0.619, Sensitivity = 0.56, Specificity = 0.68, 95%CI (0.58–0.66). PNI **(B)**: AUC = 0.665, Sensitivity = 0.60, Specificity = 0.65, 95%CI (0.62–0.71), Cut-off value = 48. Combined CFS + PNI **(C)**: AUC = 0.694, Sensitivity = 0.67, Specificity = 0.63, 95%CI (0.65–0.74). CFS, Clinical Frailty Scale; PNI, Prognostic Nutritional Index; AUC, area under the curve; CI, confidence interval.

The CFS demonstrated satisfactory predictive value for postoperative complications, with an AUC of 0.619 (95%CI 0.575–0.662), and the sensitivity and specificity of 0.56 and 0.68, respectively. Similarly, the PNI showed satisfactory predictive accuracy with an AUC of 0.665 (95%CI: 0.618–0.712), with the sensitivity and specificity of 0.60 and 0.65, respectively. As shown in the ROC curve for predicted postoperative complications, the cutoff value for PNI according to Youden’s index was established at 48. The combination of the CFS and PNI showed good predictive ability for postoperative complications, with an AUC of 0.694 (95%CI: 0.647–0.740) (Figure 1). The difference between CFS and PNI was not significant (*D* = −1.615, *p* = 0.106), nor was that between PNI and the combined model (*D* = −1.857, *p* = 0.063). However, the combined CFS and PNI model showed a significantly higher AUC compared to the CFS alone (*D* = −4.515, *p* < 0.001).

## 4 Discussion

Our study explored the integration of frailty and nutritional assessments to predict postoperative complications in older patients who underwent intermediate- to high-risk non-cardiac surgery. These findings demonstrate that both frailty and nutritional status are associated with an increased risk of postoperative complications, albeit to different extents.

### 4.1 Frailty as a predictor of postoperative complications

Frailty, as defined by the CFS, emerged as a strong independent predictor of postoperative complications in our study. This finding aligns with a growing body of evidence highlighting the significance of frailty in surgical outcomes (23–27). Moreover, the CFS has been highlighted as a valuable tool for identifying high-risk individuals (28–30). For instance, Hewitt et al. (8) reported in their systematic review and meta-analysis that frailty was associated with increased mortality, postoperative complications, and prolonged LOS in general surgery patients. Our results corroborate these findings and emphasize the robustness of frailty as a predictor in different surgical populations. In contrast, Czajka et al. (22) found that frailty assessment using CFS did not predict the occurrence of in-hospital postoperative complications. These findings highlight the potential variability in the predictive performance of the CFS across different patient populations and clinical settings. Such variability underscores the importance of context-specific validation of frailty tools prior to their routine clinical application.

The independent association between frailty and postoperative complications, even after adjusting for other covariates, underscores the importance of incorporating frailty assessment into preoperative risk stratification protocols. This is particularly relevant, given that frailty captures a range of physiological deficits that may not be apparent in routine preoperative evaluations. Fried et al. noted that frailty represents a transition from homeostatic stability to increased vulnerability to stressors (31), which may explain the strong predictive power of frailty for surgical outcomes.

### 4.2 Nutritional status and postoperative outcomes

Our analysis of nutritional status, as measured using the PNI, revealed a nuanced relationship concerning postoperative complications. While a PNI score <40—indicating severe malnutrition—showed a strong association with complications in the univariate analysis, this relationship was not significant in the multivariate model. This finding weakens the argument that malnutrition is an independent predictor of adverse outcomes in this population.

While PNI is a useful indicator of overall nutritional status and has been shown to correlate with postoperative systemic inflammatory response (32), its predictive value may be limited in the context of noninfectious postoperative complications (33). These limitations highlight the importance of interpreting PNI in conjunction with other clinical factors and support the need for further research to clarify its role in comprehensive preoperative risk assessment.

This finding contrasts with those of some previous researchers, such as Kanda et al. (19), who found that a low preoperative PNI score was significantly associated with postoperative complications. Moreover, low preoperative PNI score has been significantly associated with a higher incidence of ischemic stroke in patients undergoing non-cardiac surgery (34). Liu et al. (18) found that patients with moderate to severe malnutrition (PNI scores of 40–45) (OR: 2.92; 95%CI: 1.31–6.50) and serious malnutrition (PNI score <40) (OR: 3.15; 95%CI: 1.12–8.83) were more likely to develop postoperative delirium. In their study, the cutoff value of PNI was 46.05, determined by ROC curve analysis, and the AUC was 0.69 (95%CI: 0.62–0.77) (18). Our study found that the AUC of PNI for predicting postoperative complications was 0.665, with the cutoff value for PNI established at 48, which aligned closely with previous findings. Su et al. (27) reported an AUC of 0.705 for the Nutritional Risk Screening 2002 Scale in predicting postoperative complications, which surpassed the metrics observed in our study. Nonetheless, we observed a trend toward increased complication rates in patients with PNI scores <46, reinforcing the potential benefit of nutritional optimization before surgery.

### 4.3 Synergistic effect of integrated frailty and nutritional assessment

A key finding of our study was the improved predictive accuracy achieved by combining frailty and nutritional assessments. Although the AUC of the combined model (CFS + PNI: 0.694) did not reach the commonly accepted threshold for strong clinical discrimination (AUC ≥ 0.7), it was significantly higher than that of the CFS alone (AUC = 0.619, *p* < 0.001), suggesting that adding nutritional assessment may improve risk stratification beyond frailty alone. These findings support the value of a multidimensional assessment approach, incorporating both functional and nutritional domains, in preoperative risk evaluation. However, the moderate overall predictive power and overlapping variance may reflect shared underlying mechanisms—such as inflammation, sarcopenia, and diminished physiological reserve—which should be further investigated.

The synergistic effect of frailty and malnutrition likely arises from overlapping and complementary mechanisms. Frailty reflects systemic physiological decline, reducing the body’s ability to cope with surgical stress (35), while malnutrition impairs cellular repair mechanisms and immune function, delaying recovery (36). For instance, malnutrition exacerbates sarcopenia—a hallmark of frailty—further reducing muscle strength and resilience. Our findings align with those of prior studies. For example, Su et al. (27) found that combining frailty with nutritional risk assessment could increase the predictive power of postoperative complications in older patients with gastrointestinal malignancies (AUC = 0.844). In comparison, our combined model yielded a more modest AUC of 0.694. The differences in predictive performance may be attributed to variations in study populations, nutritional assessment tools, and definitions of postoperative outcomes. This synergistic effect suggests that frailty and nutritional status capture distinct yet complementary aspects of a patient’s overall health and resilience to surgical stress.

The coexistence of frailty and malnutrition in 16.6% of our study population aligns with previous reports indicating a range of 8–33% (37–41) suggesting a consistent pattern across various older populations. This overlap highlights the importance of comprehensive geriatric assessment during preoperative evaluation.

Our findings align with those of a recent study by Khajoueinejad et al. (42), who reported that preoperative frailty and malnutrition in surgical oncology patients predicted higher postoperative adverse events and worse survival. The superior predictive performance of our combined assessment model underscores its potential to enhance preoperative risk stratification through a comprehensive evaluation approach.

These findings suggest that routine preoperative assessments of both frailty and nutritional status may help in identifying high-risk older patients. Interventions targeting these factors may improve postoperative outcomes; however, further research is required to confirm this result.

### 4.4 Strengths and limitations

A key strength of our study was that it included both frailty and nutritional status, which provided a more holistic understanding of the factors influencing postoperative outcomes in older patients. This dual approach helps capture the key elements that affect patient vulnerability in the surgical setting. Moreover, the relatively large sample size strengthened the statistical power of the analysis and enhanced the generalizability of the findings to other older surgical populations. This study had some limitations that should be considered when interpreting our findings. First, the observational nature of the study precludes causal inferences between frailty, nutritional status, and postoperative outcomes. Additionally, although we adjusted for multiple covariates, residual confounding factors could not be ruled out. Finally, our sample was limited to a single tertiary care center, which may limit the generalizability of our findings to other healthcare settings or populations with different demographic and clinical characteristics.

In conclusion, our study demonstrated that frailty is a robust predictor of postoperative complications in older patients undergoing non-cardiac surgery, while the impact of malnutrition may be more complex. The improved predictive accuracy achieved by combining frailty and nutritional assessments underscores the potential value of comprehensive geriatric evaluation in preoperative risk stratification. These findings contribute to the growing evidence supporting the integration of geriatric-specific measures into surgical care pathways for older adults, potentially leading to improved patient outcomes and optimizing the allocation of healthcare resources.

## Data Availability

The datasets presented in this study can be found in online repositories. The names of the repository/repositories and accession number(s) can be found in the article/Supplementary material.
