# Supplementary material for: Frailty and nutritional assessments for predicting postoperative complications in older adults undergoing non-cardiac surgery
Source: Front Med (Lausanne). 2025 Aug 6;12:1636091. doi: 10.3389/fmed.2025.1636091 (PMC12364851; doi:10.3389/fmed.2025.1636091)
Supplement: Supplementary file 1 [file Data_Sheet_1.pdf]

Supplementary Table S1. Demographic and clinical characteristics stratified by CFS and PNI in older patients undergoing intermediate- to high-risk non-cardiac surgery

	CFS				PNI				
	**Robust (n=38)**	**Pre-frail (n=354)**	**Frail (n=245)**	**p value**	**Serious malnutrition (PNI < 40, n=62)**	**Moderate to severe malnutrition (PNI 40–45, n=128)**	**Mild malnutrition (PNI 45–50, n=196)**	**Normal (PNI >50, n=251)**	**p value**
**Age** (years), median (IQR)	67 (62.2–70.8)	68 (64–73)	73 (67–79)	<0.001	74 (67.2–78.8)	71 (65–77)	70 (65–75)	68 (64–72.5)	<0.001
**Sex (**male), n (%)	17 (44.7)	194 (55.1)	95 (38.8)	<0.001	34 (54.8)	68 (53.5)	105 (53.6)	99 (39.6)	0.007
**BMI (**kg/m^2), median (IQR)	25.6 (21.6–28.7)	24 (21.6–27.2)	23.4 (20.4–26.6)	0.014	21.6 (19.3–25.8)	22.4 (19.7–25.8)	23 (21–25.4)	25.7 (22.8–28.5)	<0.001
**Marital status**, n (%)				0.001					0.76
-married or common law	30 (78.9)	281 (79.6)	161 (66)		45 (73.8)	95 (74.2)	139 (70.9)	193 (77.2)	
-never married	3 (7.9)	15 (4.2)	8 (3.3)		3 (4.9)	3 (2.3)	8 (4.1)	12 (4.8)	
-widowed	3 (7.9)	41 (11.6)	58 (23.8)		11 (18)	21 (16.4)	36 (18.4)	34 (13.6)	
-divorced/separated	2 (5.3)	16 (4.5)	17 (7)		2 (3.3)	9 (7)	13 (6.6)	11 (4.4)	
**Education**, n (%)				<0.001					0.007
-less than high school	13 (34.2)	165 (46.6)	168 (68.9)		39 (62.9)	80 (63)	109 (55.6)	118 (47)	
-high school	9 (23.7)	92 (26)	46 (18.9)		16 (25.8)	26 (20.5)	47 (24)	58 (23.1)	
-≥bachelor degrees	16 (42.1)	97 (27.4)	30 (12.3)		7 (11.3)	21 (16.5)	40 (20.4)	75 (29.9)	
**Living situation**				<0.001					< 0.001
-living at home independently	10 (26.3)	128 (36.2)	184 (75.1)		30 (48.4)	75 (58.6)	99 (50.5)	118 (47)	
-living at home with help	1 (2.6)	4 (1.1)	31 (12.7)		11 (17.7)	11 (8.6)	9 (4.6)	5 (2)	
-live alone	27 (71.1)	222 (62.7)	30 (12.2)		21 (33.9)	42 (32.8)	88 (44.9)	128 (51)	

	CFS				PNI				
	**Robust (n=38)**	**Pre-frail (n=354)**	**Frail (n=245)**	**p value**	**Serious malnutrition (PNI < 40, n=62)**	**Moderate to severe malnutrition (PNI 40–45, n=128)**	**Mild malnutrition (PNI 45–50, n=196)**	**Normal (PNI >50, n=251)**	**p value**
**Comorbidities**, n (%)	32 (84.2)	332 (93.8)	232 (94.7)	0.048	61 (98.4)	119 (93)	187 (95.4)	229 (91.2)	0.12
- Ischemic heart disease	1 (3.1)	34 (10.1)	28 (12.1)	0.285	9 (14.5)	12 (9.4)	20 (10.2)	22 (8.8)	0.592
- Heart failure	0 (0)	6 (1.8)	4 (1.7)	1	2 (3.2)	0 (0)	4 (2)	4 (1.6)	0.265
- Hypertension	7 (21.9)	214 (63.9)	173 (74.6)	<0.001	43 (69.4)	72 (56.2)	126 (64.3)	153 (61)	0.289
- Dyslipidemia	10 (31.2)	212 (63.5)	152 (65.5)	<0.001	33 (53.2)	61 (47.7)	129 (65.8)	151 (60.2)	0.009
- Diabetes	1 (3.1)	73 (21.9)	54 (23.3)	0.032	11 (17.7)	29 (22.7)	33 (16.8)	55 (21.9)	0.467
- Chronic lung disease	1 (3.1)	19 (5.7)	19 (8.2)	0.367	7 (11.3)	5 (3.9)	18 (9.2)	9 (3.6)	0.019
- CKD stage more than or equal to 3	1 (3.1)	40 (12)	31 (13.4)	0.25	11 (17.7)	19 (14.8)	23 (11.7)	19 (7.6)	0.053
- CVD	2 (6.2)	24 (7.2)	30 (12.9)	0.061	11 (17.7)	10 (7.8)	18 (9.2)	17 (6.8)	0.054
- Cancer	23 (71.9)	160 (48.2)	84 (36.2)	<0.001	33 (53.2)	55 (43)	88 (44.9)	91 (36.3)	0.062
- Metastasis	11 (47.8)	57 (35.4)	38 (45.2)	0.228	16 (25.8)	24 (18.8)	36 (18.4)	30 (12)	0.036
**Visual impairment**, n (%)	17 (44.7)	227 (64.1)	142 (58)	0.038	33 (53.2)	76 (59.4)	130 (66.3)	147 (58.6)	0.202
**Hearing impairment**, n (%)	7 (18.4)	120 (33.9)	79 (32.2)	0.153	23 (37.1)	41 (32)	77 (39.3)	65 (25.9)	0.021
**ASA class, median (IQR)**	2 (2–2)	2 (2–3)	3 (2–3)	<0.001	3 (2–3)	3 (2–3)	2 (2–3)	2 (2–3)	<0.001
**RCRI**, n (%)				0.579					0.007
- 0	29 (76.3)	208 (58.8)	150 (61.2)		33 (53.2)	71 (55.5)	110 (56.1)	173 (68.9)	
- 1	8 (21.1)	120 (33.9)	75 (30.6)		24 (38.7)	51 (39.8)	64 (32.7)	64 (25.5)	
- ≥ 2	1 (2.6)	22 (6.2)	16 (6.5)		5 (8.1)	6 (4.7)	22 (11.2)	14 (5.6)	

	CFS				PNI				
	**Robust (n=38)**	**Pre-frail (n=354)**	**Frail (n=245)**	**p value**	**Serious malnutrition (PNI < 40, n=62)**	**Moderate to severe malnutrition (PNI 40–45, n=128)**	**Mild malnutrition (PNI 45–50, n=196)**	**Normal (PNI >50, n=251)**	**p value**
**Cardiac risk stratification surgical procedure**, n (%)				0.021					0.027
- High-risk (172)	9 (23.7)	111 (31.4)	52 (21.3)		16 (25.8)	37 (28.9)	66 (33.8)	53 (21.2)	
- Intermediate-risk (463)	29 (76.3)	242 (68.6)	192 (78.7)		46 (74.2)	91 (71.1)	129 (66.2)	197 (78.8)	
**Preoperative anemia**, n (%)	17 (44.7)	188 (53.1)	180 (73.5)	<0.001	55 (88.7)	102 (79.7)	121 (61.7)	107 (42.6)	<0.001
**Albumin level**, median (IQR)	4 (3.7–4.3)	4 (3.8–4.3)	3.8 (3.5–4.1)	<0.001	3 (2.7–3.3)	3.5 (3.4–3.7)	3.9 (3.8–4.1)	4.2 (4–4.4)	<0.001
**PNI**, median (IQR)	51.5 (47–54.8)	50 (46–53)	46 (42–51)	<0.001					
**CrCl**, median (IQR)	71.8 (62–80.8)	62.7 (49–77)	58 (43.4–76)	0.004	56.5 (42–75.2)	59 (40.8–76.6)	59 (48–75)	68 (54–80.1)	<0.001
**Mid-arm circumference (cm); median** (IQR)	27.8 (25.2–29.9)	27 (24.5–29)	26.5 (24–29)	0.133	24 (22–26.9)	25.5 (22.9–27)	26.8 (24.5–28.6)	28 (26–30)	<0.001
**Frail**, n (%)					38 (61.3)	68 (53.1)	69 (35.2)	70 (27.9)	<0.001

Abbreviations: ASA, American Society of Anesthesiologists; BMI, body mass index; CFS, Clinical Frailty Scale; CKD, chronic kidney disease; CrCl, creatinine clearance; CVD, cerebrovascular disease; IQR, interquartile range; PNI, Prognostic Nutritional Index; RCRI, Revised Cardiac Risk Index
