# Supplementary material for: Frailty and nutritional assessments for predicting postoperative complications in older adults undergoing non-cardiac surgery
Source: Front Med (Lausanne). 2025 Aug 6;12:1636091. doi: 10.3389/fmed.2025.1636091 (PMC12364851; doi:10.3389/fmed.2025.1636091)
Supplement: Supplementary file 2 [file Data_Sheet_2.pdf]

Supplementary Table S2. Postoperative complications and outcomes stratified by CFS and PNI in older patients undergoing intermediate- to high-risk non-cardiac surgery

Postoperative complications and Outcomes	CSF			PNI				
	Non-frail (n=392)	Frail (n=245)	p value	Serious malnutrition Score of < 40 (n=62)	Moderate to severe malnutrition Score of 40–45 (n=128)	Mild malnutrition Score of 45–50 (n=196)	Normal Score of >50 (n=251)	p value
Overall postoperative complications, n (%)	87 (22.2)	110 (44.9)	<0.001	37 (59.7)	46 (35.9)	67 (34.2)	47 (18.7)	<0.001
Cardiac complication, n (%)	25 (27.8)	16 (14.5)	0.033	7 (18.4)	7 (15.2)	16 (23.2)	11 (23.4)	0.697
Pulmonary complication, n (%)	36 (40)	31 (28.2)	0.107	15 (39.5)	13 (28.3)	24 (34.8)	15 (31.9)	0.734
Neurologic complication, n (%)	6 (6.7)	15 (13.6)	0.18	4 (10.8)	4 (8.7)	9 (13)	4 (8.5)	0.855
Renal complication, n (%)	18 (20.2)	21 (19.1)	0.983	9 (24.3)	5 (10.9)	13 (18.8)	12 (25.5)	0.281
Infection, n (%)	16 (18)	25 (22.7)	0.517	8 (21.6)	14 (30.4)	12 (17.4)	7 (14.9)	0.249
Multiorgan failure, n (%)	1 (1.1)	1 (0.9)	1	0 (0)	1 (2.2)	0 (0)	1 (2.1)	0.424
Transfusion of blood products, n (%)	26 (29.2)	64 (58.2)	<0.001	19 (51.4)	24 (52.2)	33 (47.8)	14 (29.8)	0.104
Use vasopressor or inotrope, n (%)	4 (4.5)	9 (8.2)	0.448	2 (5.4)	3 (6.5)	6 (8.7)	2 (4.3)	0.831
Unplanned return to OR, n (%)	3 (3.4)	15 (13.6)	0.024	7 (18.9)	5 (10.9)	3 (4.3)	3 (6.4)	0.093
Unplanned return to ICU, n (%)	5 (5.6)	5 (4.5)	0.755	2 (5.4)	1 (2.2)	4 (5.8)	3 (6.4)	0.804
Duration of surgery (mins), median (IQR)	252.5 (175–370)	260 (180–350)	0.907	325 (225–435)	305 (188.8–411.2)	240 (183.8–345)	225 (167.5–325)	<0.001
Blood loss (mL), median (IQR)	100 (20–300)	150 (50–400)	<0.001	150 (50–500)	200 (50–500)	100 (50–300)	50 (20–200)	<0.001
The Clavien-Dindo classification, median (IQR)	1 (1–1)	1 (1–3)	<0.001	1 (1–3.8)	1 (1–3)	1 (1–2)	1 (1–1)	<0.001

Postoperative complications and Outcomes	CSF			PNI				
	**Non-frail (n=392)**	**Frail (n=245)**	**P value**	**Serious malnutrition Score of < 40 (n=62)**	**Moderate to severe malnutrition Score of 40–45 (n=128)**	**Mild malnutrition Score of 45–50 (n=196)**	**Normal Score of >50 (n=251)**	**p value**
Clavien-Dindo classification ≥2, n (%)	73 (18.6)	93 (38)	<0.001	30 (48.4%)	47 (36.7)	54 (27.6)	35 (13.9)	<0.001
30-day mortality, n (%)	2 (0.5)	5 (2)	0.114	2 (3.2)	2 (1.6)	0 (0)	3 (1.2)	0.106
Death during admission, n (%)	1 (0.3)	3 (1.2)	0.161	2 (3.2)	1 (0.8)	0 (0)	1 (0.4)	0.049
Hospital LOS (day), median (IQR)	6 (4–9)	7 (5–11)	<0.001	9 (7–17.5)	8 (5–12.2)	6 (4–9)	5 (4–7)	<0.001
ICU LOS (day), median (IQR)	0 (0–0)	0 (0–0)	0.09	0 (0–0.8)	0 (0–0)	0 (0–0)	0 (0–0)	<0.001
30-day readmission, n (%)	17 (4.3)	19 (7.8)	0.101	4 (6.5)	13 (10.2)	8 (4.1)	11 (4.4)	0.085

Abbreviations: CFS, Clinical Frailty Scale; ICU, intensive care unit; IQR, Interquartile range; LOS, length of stay; OR, Operating room; PNI, Prognostic Nutritional Index
